# Marker enzyme activities in hindleg from creatine-deficient AGAT and GAMT KO mice – differences between models, muscles, and sexes

**DOI:** 10.1038/s41598-020-64740-8

**Published:** 2020-05-14

**Authors:** Karina Barsunova, Marko Vendelin, Rikke Birkedal

**Affiliations:** Laboratory of Systems Biology, Department of Cybernetics, Tallinn University of Technology, Tallinn, Estonia

**Keywords:** Biochemistry, Physiology, Skeletal muscle

## Abstract

Creatine kinase (CK) functions as an energy buffer in muscles. Its substrate, creatine, is generated by L-arginine:glycine amidinotransferase (AGAT) and guanidinoacetate N-methyltransferase (GAMT). Creatine deficiency has more severe consequences for AGAT than GAMT KO mice. In the present study, to characterize their muscle phenotype further, we recorded the weight of tibialis anterior (TA), extensor digitorum longus (EDL), gastrocnemius (GAS), plantaris (PLA) and soleus (SOL) from creatine-deficient AGAT and GAMT, KO and WT mice. In GAS, PLA and SOL representing glycolytic, intermediate and oxidative muscle, respectively, we recorded the activities of pyruvate kinase (PK), lactate dehydrogenase (LDH), citrate synthase (CS) and cytochrome oxidase (CO). In AGAT KO compared to WT mice, muscle atrophy and differences in marker enzyme activities were more pronounced in glycolytic than oxidative muscle. In GAMT KO compared to WT, the atrophy was modest, differences in PK and LDH activities were minor, and CS and CO activities were slightly higher in all muscles. SOL from males had higher CS and CO activities compared to females. Our results add detail to the characterization of AGAT and GAMT KO skeletal muscle phenotypes and illustrate the importance of taking into account differences between muscles, and differences between sexes.

## Introduction

Creatine kinase (CK) is important as a temporal and spatial energy buffer that keeps an appropriate ATP/ADP ratio near ATPases in tissues with high and fluctuating energy demand, including brain, heart and skeletal muscles^1^. Skeletal muscles mainly express cytosolic MM-CK and mitochondrial, sarcomeric Mi-CK. The overall activity and isoform distribution varies between muscles, and is related to their metabolic and contractile profile^2,3^. In general, glycolytic, fast-twitch muscles have a high activity of cytosolic CK and a small fraction of Mi-CK, whereas oxidative, slow-twitch muscles have a lower total CK activity and a higher proportion of Mi-CK^3^.

The physiological role of the CK system in muscles has been studied in several loss-of-function models interfering with creatine synthesis in the body, creatine uptake, or expression of different CK isoforms. Creatine is synthesized by Arginine:Glycine amidinotransferase (AGAT, EC 2.1.4.1) and Guanidinoacetate N-methyltransferase (GAMT, EC 2.1.1.2), and mouse knockout models of both exist^4,5^. Creatine uptake can be inhibited competitively by feeding with the creatine-analogue beta-guanidinopropionic acid (β-GPA), and by knockout of the creatine transporter. Lastly, there are mice lacking the muscle-specific cytosolic (M-CK) or mitochondrial (Mi-CK) CK isoforms, or both (CK KO).

All models have in common that the skeletal muscles shift towards a more aerobic metabolism. In general, there is an increase in mitochondrial volume as well as the activity of citrate synthase, CS (marker of mitochondrial content), and cytochrome oxidase, CO (complex IV and marker of aerobic capacity)^4,6–12^. Interfering with creatine synthesis and uptake seems to affect the phenotype more than knockout of CK itself. Mi-CK KO mice have the mildest phenotype with no changes in morphology or contractility^13^. M-CK and CK KO mice have a normal body weight^9,14^. The twitch force is the same as in WT^7^, but they lack burst activity and contraction is slowed^15^. Interfering with creatine synthesis or uptake lowers the body weight and causes muscle atrophy^4,5,11,16^. In terms of body weight, atrophy and grip strength, the phenotype of GAMT KO mice is not as severe as that of AGAT KO mice^4,10,17^.

The aim of the present study was to assess in more detail the changes in skeletal muscle weight and activity of metabolic marker enzymes in hindleg muscles of creatine-deficient GAMT and AGAT KO mice. In CK KO mice, the CS and CO activities are elevated in glycolytic muscles, which rely more on CK, but not in oxidative muscles^9^. Here, we assessed whether the phenotypic changes in AGAT and GAMT KO were muscle specific and whether there were any differences between males and females. For that, we isolated and weighed the main muscles of the hindlegs, which run along the tibia: tibialis anterior (TA) and extensor digitorum longus (EDL) on the anterior side, and gastrocnemius (GAS), plantaris (PLA) and soleus (SOL) on the posterior side of the leg. In GAS, PLA and SOL representing glycolytic, intermediate and oxidative muscle, respectively, we recorded the activities of metabolic marker enzymes: Pyruvate kinase (PK), which catalyzes the last step in glycolysis, lactate dehydrogenase (LDH), which is a marker of anaerobic glycolytic capacity, and CS and CO, which are validated markers of mitochondrial content and oxidative capacity, respectively^18^.

## Results

### Morphological data

Table 1 shows the general morphological characteristics of AGAT and GAMT mice. Body weight and tibial length were significantly lower in both AGAT and GAMT KO compared to their WT littermates. The bodyweight was strongly influenced by sex as well as genotype, as males are larger than females. In AGAT mice, we also found an interaction between genotype and sex, because the difference in WT-KO bodyweight was larger in males than females.

**Table 1:** Body weight (BW), tibial length (TL) and age of AGAT and GAMT, WT and KO, males and females.

AGAT	n	BW, g	TL, cm	Age, days
WT male	7	33.0 ± 1.2	2.20 ± 0.02	199 ± 15
KO male	7	18.6 ± 0.4	2.10 ± 0.01	178 ± 9
% of WT		56	96	
WT female	14	25.0 ± 0.8	2.17 ± 0.01	214 ± 13
KO female	7	15.4 ± 0.3	2.07 ± 0.02	217 ± 20
% of WT		62	95	
**Effect**				
G		***	***	
S		***		
Int		*		
**GAMT**				
WT male	7	34.4 ± 1.9	2.21 ± 0.02	190 ± 10
KO male	8	25.3 ± 0.8	2.16 ± 0.01	190 ± 10
% of WT		74	98	
WT female	7	26.6 ± 0.6	2.18 ± 0.01	213 ± 21
KO female	6	20.0 ± 1.7	2.11 ± 0.01	187 ± 9
% of WT		75	97	
**Effect**				
G		***	***	
S		***		
Int				

Values are shown as mean ± SEM. The effects of genotype (G; WT and KO), sex (S; male and female) and their interaction (Int) obtained by analysis with a two-way Bayesian Anova are shown below the data for each strain (AGAT and GAMT). Significance notation: *10 ≤ BF < 30 strong evidence; **30 ≤ BF < 100 very strong evidence; ***BF ≥ 100 extremely strong evidence.

Table 2 shows the absolute weights of the different muscles: For all muscles in both AGAT and GAMT mice, there was a strong effect of genotype and sex. In AGAT mice, there was also a strong interaction between genotype and sex (except for SOL). The interaction between the two factors arises from the fact that genotype affects males and females differently. In the present case, the WT-KO difference in muscle weight was larger in males than in females.

**Table 2:** Hindleg muscle weights in AGAT and GAMT, WT and KO, males and females.

AGAT	n	Muscle weight, MW				
		GAS, mg	PLA, mg	SOL, mg	TA, mg	EDL, mg
WT male	7	142.1 ± 3.5	20.2 ± 0.4	10.5 ± 0.3	55.6 ± 0.9	11.8 ± 0.3
KO male	7	35.3 ± 2.0	6.7 ± 0.3	6.6 ± 0.3	16.8 ± 0.7	4.1 ± 0.2
% of WT		25	33	62	30	35
WT female	14	111.5 ± 2.1	15.3 ± 0.4	8.3 ± 0.3	45.0 ± 0.9	9.3 ± 0.2
KO female	7	26.2 ± 1.4	5.4 ± 0.2	5.4 ± 0.2	13.1 ± 0.6	3.4 ± 0.1
% of WT		24	35	65	29	37
**Effect**						
G		***	***	***	***	***
S		***	***	***	***	***
Int		***	***		***	***
**GAMT**						
WT male	7	152.0 ± 5.9	21.2 ± 1.1	11.6 ± 0.7	56.3 ± 1.8	13.2 ± 0.8
KO male	8	104.2 ± 2.2	15.1 ± 0.4	7.9 ± 0.2	36.5 ± 1.1	7.6 ± 0.3
% of WT		69	71	68	65	58
WT female	7	123.0 ± 2.9	17.3 ± 0.7	8.8 ± 0.3	46.1 ± 0.9	10.3 ± 0.3
KO female	6	78.3 ± 3.0	11.0 ± 0.4	6.6 ± 0.3	27.6 ± 1.2	5.9 ± 0.2
% of WT		64	64	75	60	57
**Effect**						
G		***	***	***	***	***
S		***	***	***	***	***
Int						

Values are shown as mean ± SEM. Statistical analysis and notation is the same as in Table 1.

Table 3 shows the muscle weights normalized to the body weight. In AGAT mice, the relative muscle weight was strongly affected by genotype in all muscles, except SOL. In GAMT mice, there was a strong effect of genotype only on the relative weight of TA and EDL.

**Table 3:** Hindleg muscle weights (MW) normalized to body weight (BW) in AGAT and GAMT, WT and KO, males and females.

AGAT	n	Relative muscle weight, MW/BW				
		GAS, mg/g	PLA, mg/g	SOL, mg/g	TA, mg/g	EDL, mg/g
WT male	7	4.33 ± 0.18	0.62 ± 0.02	0.32 ± 0.02	1.69 ± 0.06	0.36 ± 0.01
KO male	7	1.90 ± 0.08	0.36 ± 0.04	0.35 ± 0.01	0.90 ± 0.03	0.22 ± 0.01
% of WT		44	58	110	53	62
WT female	14	4.50 ± 0.12	0.62 ± 0.02	0.34 ± 0.01	1.84 ± 0.05	0.38 ± 0.01
KO female	7	1.70 ± 0.08	0.35 ± 0.01	0.35 ± 0.01	0.85 ± 0.04	0.22 ± 0.01
% of WT		38	57	105	46	59
**Effect**						
G		***	***		***	***
S						
Int						
GAMT						
WT male	7	4.44 ± 0.17	0.62 ± 0.03	0.34 ± 0.02	1.66 ± 0.07	0.39 ± 0.02
KO male	8	4.13 ± 0.06	0.60 ± 0.01	0.31 ± 0.01	1.44 ± 0.02	0.30 ± 0.00
% of WT		93	97	93	87	77
WT female	7	4.48 ± 0.18	0.62 ± 0.02	0.32 ± 0.01	1.68 ± 0.07	0.38 ± 0.02
KO female	6	3.94 ± 0.12	0.56 ± 0.02	0.33 ± 0.01	1.39 ± 0.07	0.30 ± 0.01
% of WT		88	89	104	81	79
Effect						
G					***	***
S						
Int						

Values are shown as mean ± SEM. Statistical analysis and notation is the same as in Table 1.

### Marker enzymes

**Figure 1:**
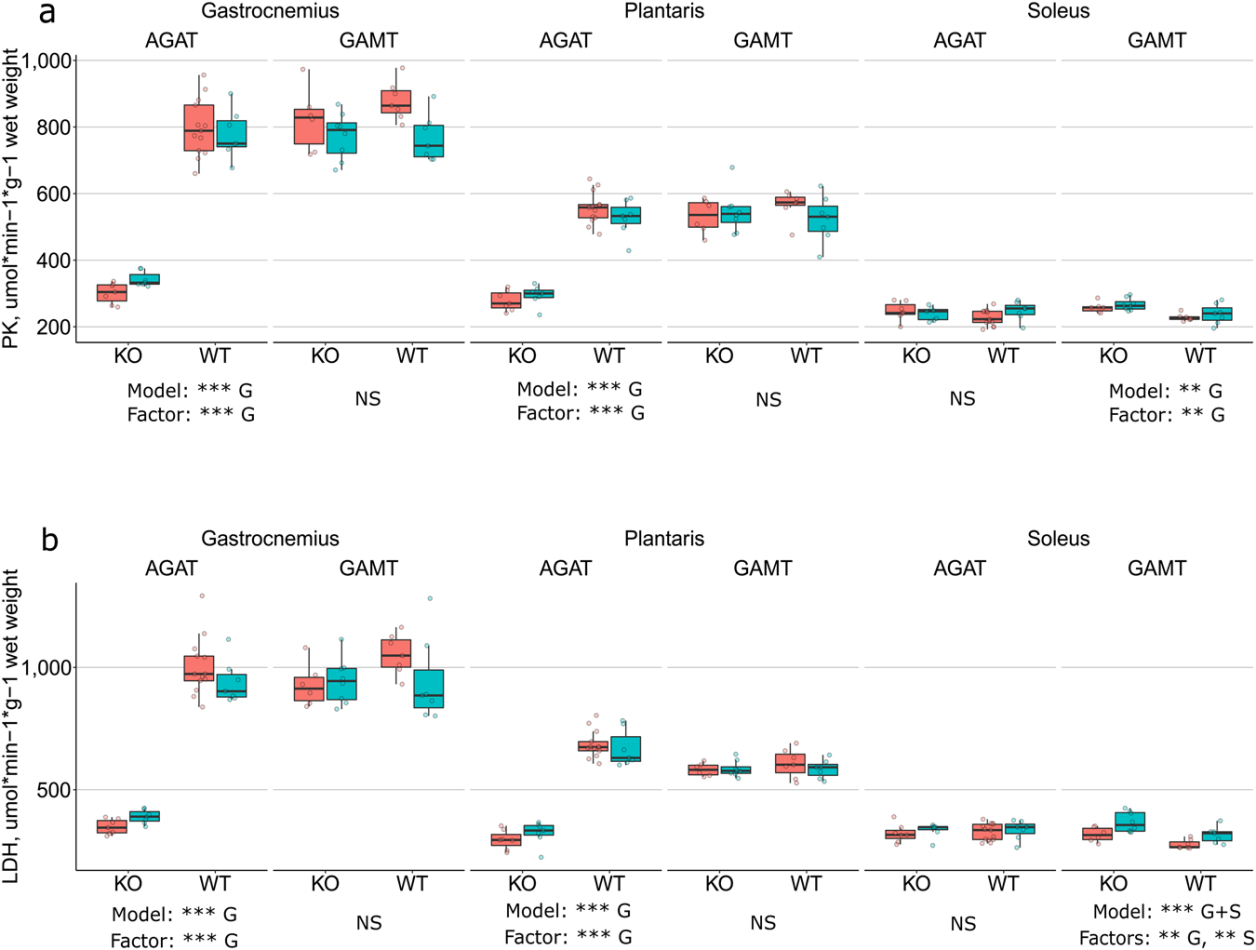
PK (**a**) and LDH (**b**) activity in GAS, PLA and SOL from AGAT and GAMT, WT and KO, males and females. The results are shown as box-and-whisker plots according to the Tukey notation as well as by individual datapoints (circles). Females are shown in orange and males are shown in turquoise. The numbers of experiments are the same as in the tables. Below each genotype for each muscle are the statistical results as determined by a two-way Bayesian Anova: the best fitting model and the significant factors within that model. G – effect of genotype (WT, KO); S – effect of sex (female, male). Significance notation: BF < 10 not significant (NS), 30 ≤ BF < 100 very strong evidence (**), and BF ≥ 100 extremely strong evidence (***).

**Figure 2:**
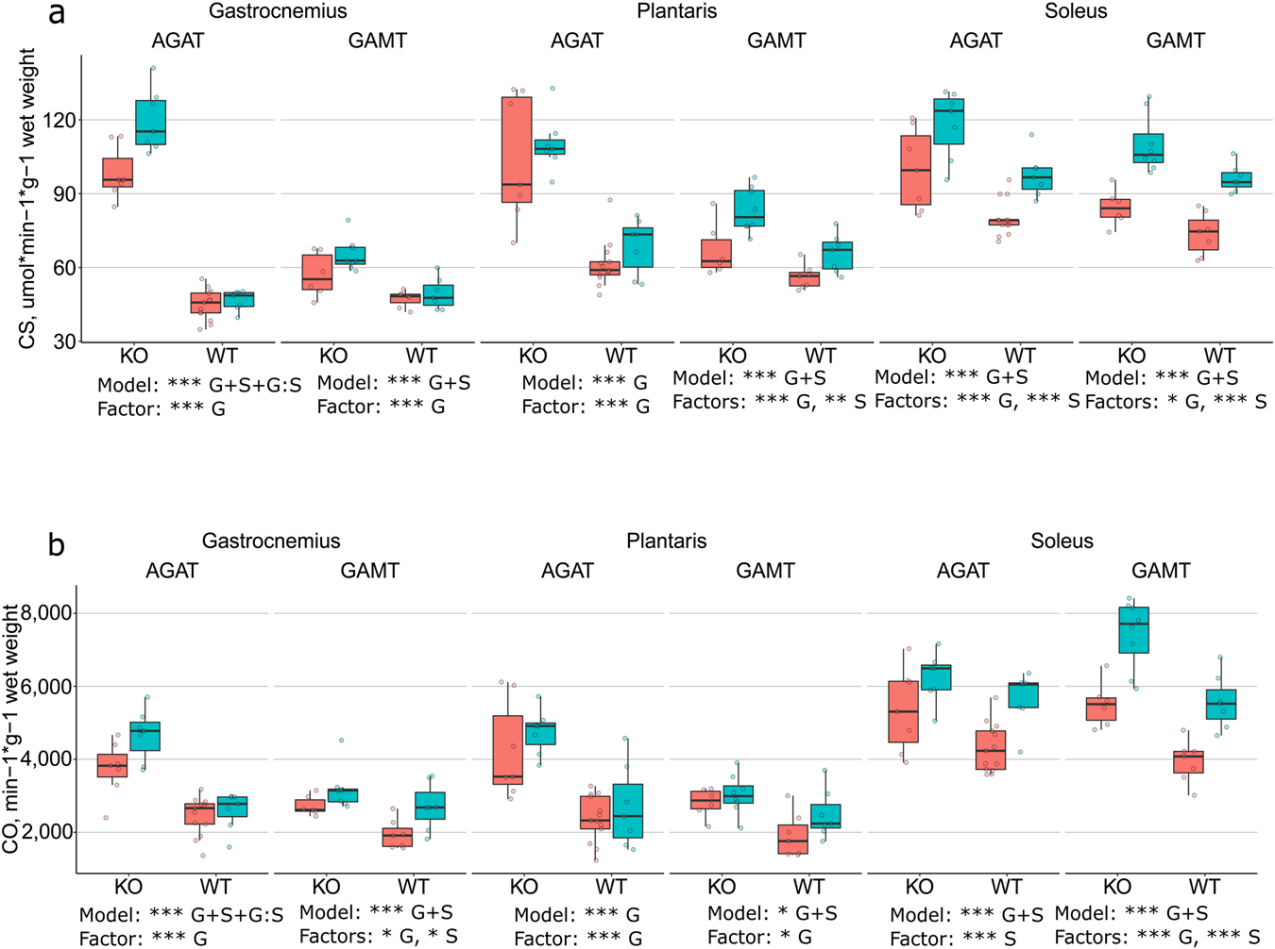
CS (**a**) and CO (**b**) activity in GAS, PLA and SOL from AGAT and GAMT, WT and KO, males and females. Notation is the same as in Fig. 1, and the numbers of experiments are the same as in the tables. Significance notation: 10 ≤ BF < 30 strong evidence (*), 30 ≤ BF < 100 very strong evidence (**), and BF ≥ 100 extremely strong evidence (***).

The activities of PK and LDH, CS and CO are shown in Figs. 1 and 2, respectively. We noticed that the overall pattern was the same for PK and LDH, and CS and CO. Therefore, these enzyme pairs are described together. Below each figure, we show the results of the statistical analysis by Bayesian two-way ANOVA. For each muscle (GAS, PLA and SOL) and each strain (AGAT and GAMT), the ANOVA assessed the effects of genotype and sex. For each analysis, we show the best fitting model. As the contribution of the factors could be different in the model, we show below the best model, which factors were significant. The results focus on the significant factors.

The PK (Fig. 1a) and LDH (Fig. 1b) activities were significantly lower in GAS and PLA from AGAT KO mice compared to their WT littermates. In GAS and PLA, the activities were ~40% and ~50%, respectively, of the activities in WT. In SOL, the PK and LDH activities did not differ between AGAT KO and WT. In GAMT mice, there was no effect of genotype in GAS and PLA, but the PK and LDH activities were ~15% higher in SOL from KO compared to WT. In GAMT SOL, there was also an effect of sex: males had a higher LDH activity than females.

The mitochondrial CS activity (Fig. 2a) was more than doubled in GAS and nearly doubled in PLA from AGAT KO mice compared to WT. The CO activity (Fig. 2b) was nearly doubled in both GAS and PLA from AGAT KO mice compared to WT. In SOL, the CS activity was affected by genotype and sex, whereas the CO activity only was affected by sex. In GAMT mice, the CS and CO activities were higher in all three muscles in KO compared to WT. In contrast to AGAT mice, the difference between KO and WT was modest and did not vary much between muscles. Sex had a significant effect on CS in PLA and SOL, and on CO in GAS and SOL. In all cases of sex differences, the muscles from males had higher CS and CO activities than those from females. In SOL from WT mice, males had ~30% higher CS and CO activity than females.

In WT mice as well as in GAMT KO mice, the enzyme activities differed between muscles (Figs. 1 and 2): GAS had the highest activities of PK and LDH, and SOL the lowest. The pattern was opposite for CS and CO, where SOL had the highest activities and GAS the lowest. PLA was in between. However, we noticed that in AGAT KO muscles, the enzyme activities in GAS, PLA and SOL were almost at the same level. A two-way Bayesian ANOVA focusing on the muscles in AGAT KO mice (assessing the effects of muscle type and sex), demonstrated that the CS activity was similar in in all three muscles (GAS, PLA and SOL) from AGAT KO, whereas the activities of PK, LDH and CO differed slightly, but significantly, between muscles.

## Discussion

Our results are in agreement with other studies showing that creatine-deficiency in AGAT and GAMT lowers the body weight^4,19^. Others have found that the CS activity was elevated in skeletal muscles from AGAT KO^10^, whereas in GAMT KO any differences were not statistically significant^4^. The present study adds more detail to the overall picture. The phenotypic changes in response to creatine deficiency vary not only between AGAT and GAMT, but also between muscles (GAS, PLA and SOL). Furthermore, we consistently found higher activities of CS and CO in male compared to female SOL muscles. Thus, our results illustrate the importance of considering differences between muscles as well as differences between males and females.

In AGAT KO mice, GAS (glycolytic muscle) was more affected than SOL (oxidative muscle). GAS exhibited the largest shift in enzyme activities (Figs. 1 and 2) and the greatest extent of atrophy. The GAS absolute and relative muscle weights were ~25% and ~40% of WT, respectively (Tables 2 and 3). In contrast, in AGAT KO SOL, there was a small shift only in the CS activity, and the smallest extent of atrophy. The SOL absolute muscle weight was ~65% of WT (Table 2), whereas its relative weight was not different from that in WT (Table 3). PLA was in between. A study on hamstring muscles in AGAT mice found a 170% higher CS activity in AGAT KO compared to WT^10^. This is close to the shift in CS activity in PLA in the present study. Indeed, in mice, the biceps femoris, which belongs to the group of hamstring muscles, is a mixed type of muscle with small fractions of type I and type IIA fibers (oxidative), and ~93% type IIB/X (glycolytic)^20^. In terms of fiber type composition, it is close to the profile of PLA^9^. Thus, our results are close to those found by others, when comparing muscles of similar fiber composition. The larger shift in glycolytic than oxidative muscles meant that the enzyme activity profile of GAS, PLA and SOL were remarkably similar in AGAT KO mice: the activities of PK, LDH and CO were close, and the CS activity was indeed the same, in all three muscles.

The changes in GAMT KO mice were modest. Their muscle weight was on average 65% of that in WT mice (ranging from 57 to 75%, Table 2). When muscle weight was normalized to the body weight, there was no significant difference between KO and WT in GAS, PLA and SOL (Table 3). PK and LDH activities in GAS and PLA were the same as in WT, and SOL had ~15% higher activities (Fig. 1). All GAMT KO muscles exhibited ~20% higher CS and ~40% higher CO activities than GAMT WT (Fig. 2). The phenotype of GAMT KO mice is close to that of CK KO mice, which exhibit a similar extent of atrophy and no change in muscle LDH activity. CK KO mice exhibited a larger shift in CS and CO activities in GAS than in SOL^9^, and there was a similar trend in GAMT KO.

Others have shown that GAMT KO mice exhibit a milder phenotype than AGAT KO mice in terms of body weight, muscle atrophy and grip strength^4,10,17^. The myocyte diameter is smaller in both AGAT and GAMT KO compared to WT, but tends to be even smaller in AGAT than in GAMT KO^17^. The present results are in line with this. Both the absolute and relative muscle weights were less reduced in GAMT than in AGAT KO (Tables 2 and 3). Marker enzyme activities in GAS and PLA were also less affected in GAMT compared to AGAT KO mice (Figs. 1 and 2). This raises questions about the interaction between CK and AMP activated protein kinase (AMPK), which regulates the metabolism (anabolism/catabolism) according to the cellular energy levels^21^. The skeletal muscle phenotype of AGAT KO mice is directly related to creatine-deficiency, as it is rescued by creatine supplementation^10^. It was shown that in AGAT KO skeletal muscle (but not cardiac muscle^22^), AMPK is chronically active^19^. AMPK stimulates mitochondrial biogenesis through several downstream effectors, one of which is PGC1α^21^. Indeed, AGAT KO skeletal muscle also exhibit increased expression of PGC1α and cytochrome oxidase^23^. PGC1α has been termed a master regulator of mitochondrial biogenesis, and its overexpression converts fast, glycolytic muscle to slow, oxidative muscle^24^. The higher PGC1α expression in AGAT KO muscles may be one explanation why GAS, PLA and SOL in AGAT KO mice have similar, high CS activities (Fig. 2a). PGC1α overexpression is not associated with atrophy^24^, and the severe atrophy of AGAT KO muscles is likely related to the overall role of AMPK as an inhibitor of anabolic pathways^21^. The more severe phenotype of AGAT KO mice could relate to the fact that they are a model of pure creatine deficiency^10^, whereas GAMT KO mice are not. GAMT KO mice accumulate guanidinoacetate^5^, which functions as a substrate for cytosolic CK^25^ albeit at a 100 times slower reaction rate^26^. It is conceivable that the milder phenotype of GAMT KO muscles is due to that CK reacting with guanidinoacetate prevents activation of AMPK and the associated increase in PGC1α and mitochondrial biogenesis. Any direct interaction between creatine kinase and AMPK is controversial. It was suggested by one study^27^, but this was subsequently challenged by others^28,29^. It is, however, a complex topic, as the regulation of AMPK depends on tissue and subcellular localization^30^. Creatine kinase and AMPK may still interact indirectly: Creatine kinase buffers the phosphorylation potential, which, through the action of adenylate kinase, affects the level of AMP. AMP stimulates AMPK allosterically as well as by inhibiting its dephosphorylation^29^. The tissue-specific interactions between CK and AMPK and how they grade with CK activity deserve further studies.

The importance of taking into account differences between males and females is increasingly being recognized^31,32^. Males have a larger body weight (Table 1) as well as muscle mass (Table 2) than females. In the present study, we consistently found an effect of sex on the activities of CS and CO in SOL from both AGAT and GAMT mice. There was also an effect of sex on CS activity in GAMT PLA and on CO activity in GAMT GAS (Fig. 2). In all cases, the activities were higher in male than in female muscles. This is intriguing, because there seems to be a general consensus that females have a higher aerobic capacity, fatigue-resistance and proportion of slow myosin isoforms^33^. Indeed, some studies suggest this also to be the case in mice^34–36^. However, one study found that hindlimb muscles from males and females had similar CS and CO activities^37^. Another study suggested, similar to the present, that sex differences are muscle specific, and that CS activity in SOL was lower in females than in males^38^. As sex differences in muscle phenotype vary between studies, species and muscles within a species, more studies are needed in this area.

In conclusion, we have reported the muscle weight and the activities of PK, LDH, CS and CO in GAS, PLA and SOL from creatine-deficient AGAT and GAMT KO mice compared to their WT littermates. The KO-WT differences vary between muscles. In addition, in SOL, males consistently had higher activities of CS and CO than females. Our results thus illustrate the importance of taking into account differences between muscles as well as differences between males and females, when characterizing phenotypes.

## Methods

### Ethics

All animal procedures were carried out according to directive 2010/63/EU of the European Parliament and had been approved by the Project Authorization Committee for Animal Experiments in the Estonian Ministry of Rural Affairs.

### Animals

GAMT and AGAT heterozygous mice on a pure C57BL/6 J genetic background (backcrossed for > 10 generations) were originally generated by the group of Dr. Isbrandt^4,19^. We received them from The Wellcome Trust Centre for Human Genetics (Oxford, UK). The animals were kept and bred in the animal facility of Tallinn University of Technology at an ambient temperature of 22–22.8 °C and a 12:12 hours light:dark cycle. They had free access to water and food (naturally creatine-free V1534-000 Rat/mouse maintenance from Ssniff Spezialdiäten GmbH, Germany). As the AGAT KO mice were small and weak, they were housed in groups whenever possible, given moistened food at the bottom of the cage, and had longer cages with a heating lamp at one end to allow for behavioral thermoregulation. Breeding was performed with heterozygous mating to obtain KO and WT littermates. Both GAMT and AGAT KO mice were housed separately from their WT and heterozygous littermates to avoid accumulating creatine via coprophagia^4^.

The genotype of the animals was determined as described in^39^ for GAMT mice. The same protocol was used for AGAT mice, except that the following primers were used: 5′-AGCCCCTCTATTTCCCTTTTCATT-3′, 5′-TTCCACTGCGTCATTCTCCTGTAA-3′. Furthermore, for AGAT samples, the touchdown PCR protocol was as follows: An initial denaturation at 95 °C for 5 min was followed by 10 cycles at 94 °C for 60 s, 65 °C (0.5 °C decrease per cycle) for 60 s, 72 °C for 30 sec, then another 35 cycles at 95 °C for 60 s, 60 °C for 60 s, 72 °C for 30 sec, and a final extension step was carried out at 72 °C for 2 min. Amplification of a single 522-bp product or a 277-bp PCR product corresponded to AGAT WT or AGAT KO genotype, respectively. Simultaneous amplification of a 522- and 277-bp fragments corresponded to a heterozygous AGAT genotype.

### Muscle samples

Mice, which had not been used for other experiments before, were anaesthetized with ketamine/dexmedetomidine mixture (150 mg·kg^−1^ and 0.5 mg·kg^−1^, respectively) and received an injection of heparin (250U) to prevent blood coagulation. When the toe-pinch reflex was absent, the animal was killed by cervical dislocation. The following hindleg muscles were excised: gastrocnemius, plantaris, soleus, tibialis anterior, extensor digitorum longus. They were kept in ice-cold Ringer’s solution until they were blotted and weighed, and then stored in cryovials at −80 °C until further experiments.

### Homogenization

Marker enzyme activities were recorded in GAS, PLA and SOL, representing glycolytic, intermediate and oxidative muscle, respectively. All steps of the homogenization procedure took place on ice. Each tissue sample was cut into smaller pieces with a scissor, transferred to a glass homogenizer, and homogenization buffer was added to a concentration of 50 mg tissue/ml buffer. The buffer contained HEPES (5 mM), EGTA (1 mM), Triton X-100 (0.1%), DL-Dithiothreitol (1 mM), and cOmplete Mini Protease Inhibitors (Roche, Merck). The pH was adjusted to 8.7 with KOH. The pestle was attached to a drill, and the tissue was ground until the solution was homogenous. The homogenized samples were incubated on ice for one hour before use. Fresh, non-diluted homogenates were used to measure CO activity. The remaining homogenate was kept at −80 °C until the activities of LDH, PK and CS were measured (within a few days).

### Spectrophotometer recordings

Enzyme activities were recorded in a Thermo Scientific Evolution 600 UV-Visible Spectrophotometer equipped with a Peltier water cooled cell changer (SPE 8 W, Thermo Fisher Scientific) to maintain temperature at 37 °C. During the experiments, the assay buffer in use was kept in a dry bath at 37 °C (Star Lab, model: N24004001). Preliminary recordings with a thermometer probe (CL-100, Warner Instruments) had shown that heating through the cell changer alone was insufficient. But when the assay buffer was kept at 37 °C, the temperature was stable at 37 °C before and after the reaction in the spectrophotometer. All samples were measured in triplicates.

CO activity was measured at 550 nm, which is the spectral absorption spectra peak of reduced, but not oxidized, cytochrome c. The protocol originates from^40^. The reaction took place in 1 ml sodium phosphate buffer (13 mM, pH 7.4) containing cytochrome c (0.4 mg/ml), which had been reduced with Na-dithionite. After recording the initial absorbance for 10–20 seconds, the reaction was initiated by the addition of 5–15 µl of undiluted homogenate (sample volume depended on the muscle phenotype). The reaction of cytochrome c oxidation represents a first-order reaction with respect to reduced cytochrome c and is observed as a logarithmical decline in absorption (as a function of time). The rate constant, obtained by fitting to the equation δ(Absorption) / δ(time) = k (Absorption), was normalized to the tissue wet weight, min^−1^·g^−1^. The IOCBIO Kinetics software for fitting is open source and available at https://iocbio.gitlab.io/kinetics.

LDH and PK activities were measured through the reduction of NADH to NAD^+^ at 340 nm. The LDH protocol was from^41^ but with modifications. The reaction took place in 1 ml potassium phosphate buffer (40–45 mM, pH 7.5) containing NADH (0.3 mM) and Na-pyruvate (0.75 mM). The pyruvate dependency of LDH varies between isoforms^41^. For our experiments, the concentration of 0.75 mM Na-pyruvate was chosen, because preliminary experiments, where Na-pyruvate was varied between 0.05–5 mM, showed this to be where both SOL and GAS had their maximal rate. The PK assay was based on^40^. The activity was measured in 1 ml potassium phosphate buffer (40–45 mM, pH 7.5) containing NADH (0.3 mM), PEP (4 mM), ADP (7 mM) and LDH (2.6 IU). For both assays, the reaction was initiated by the addition of 15, 10, or 5 μl of a 1:10 dilution of SOL, PLA and GAS homogenates, respectively. The linear reaction rate was converted to enzyme activity using the extinction coefficient of NADH (6220 Abs·litre·mol^−1^·cm^−1^ at 340 nm) and the activity was normalized to the tissue wet weight, μmol·min^−1^·g^−1^.

The CS activity was measured in a coupled assay following the increase in absorbance at 412 nm, as the CS reaction releases CoA-SH, which forms 5-thio-2-nitrobenzoic acid (TNB) from 5,5′-dithiobis-2-nitrobenzoic acid (DTNB). The reaction rate was recorded as the increase in the rate of TNB formation upon addition of oxaloacetate (0.5 mM) to 1 ml reaction medium containing: Tris-HCl buffer (100 mM, pH 8.1), Acetyl CoA (0.3 mM) and 5, 10 or 15 μl of 1:10 times diluted homogenate from SOL, PLA or GAS, respectively. The linear reaction rate was converted to enzyme activity the using the TNB extinction coefficient (13800 Abs·liter·mol^−1^·cm^−1^ at 412 nm and 37 °C^42^) and normalized to the tissue wet weight, μmol·min^−1^·g^−1^.

### Statistics

The values are shown as mean ± standard error of the mean (SEM). Part of the statistical analysis was performed using the free software JASP. To assess the effects of gender and genotype, AGAT and GAMT data were analyzed in R using a Bayesian ANOVA script, which is included in the IOCBIO Kinetics software. The script used the BayesFactor R package (Richard D. Morey, CRAN). Bayesian ANOVA tests evaluated a hierarchy of models of different complexity, with the most complex model consisting of interactions between all factors studied, lower order of interactions, and the studied factors, separately. In the figures, the model with the largest odds against null model (largest Bayes Factor, BF) is shown in the statistical results (NS if null model had odds larger than assumed significant for alternative hypothesis, see below) together with the model-averaged inclusion Bayes factor, using matched models approach, as in^43^. In the tables, for simplicity, only the statistically significant factors are reported using the same analysis as done for the figures. The Bayes Factor (BF) interpretation considers common evidence categories^44^ as follows: BF < 10 not significant, 10 ≤ BF < 30 strong evidence (*), 30 ≤ BF < 100 very strong evidence (**), and BF ≥ 100 extremely strong evidence (***) for the tested hypothesis. The figures were made using the open source software RStudio (https://rstudio.com, version 1.2.5033).

## Data Availability

The data that support the findings of this study are available from the corresponding author upon reasonable request.
